# Integration of simulation-based education in anaesthesiology specialist training: Synthesis of results from an Utstein Meeting

**DOI:** 10.1097/EJA.0000000000001913

**Published:** 2023-10-19

**Authors:** Georges L. Savoldelli, Crina L. Burlacu, Marc Lazarovici, Francisco Maio Matos, Doris Østergaard

**Affiliations:** From the Division of Anaesthesia, Department of Anaesthesiology, Clinical Pharmacology, Intensive Care and Emergency Medicine. Geneva University Hospitals and Faculty of Medicine University of Geneva, Geneva, Switzerland (GLS), University College Dublin, School of Medicine, Surgery and Surgical Specialties and Department of Anaesthesia, Intensive Care and Pain Medicine, St. Vincent's University Hospital, Dublin, Ireland (CLB), Institute for Emergency Medicine and Management in Medicine, Ludwig Maximilians University Hospital, Munich, Germany (ML), Anaesthesiology Department, Centro Hospitalar e Universitário de Coimbra, CHUC, Coimbra, Portugal; Faculty of Medicine, University of Coimbra, FMUC, Coimbra, Portugal and Clinic Academic Center of Coimbra, CACC, Coimbra, Portugal (FMM), Copenhagen Academy for Medical Education and Simulation, Capital Region of Denmark and Faculty of Medicine, University of Copenhagen (DO), European Society of Anaesthesiology and Intensive Care (ESAIC) Simulation Committee, https://www.esaic.org/about/committees/simulation-committee/ (GLS, CLB, FMM, DO), Society for Simulation in Europe (SESAM) Executive Committee, https://www.sesam-web.org (ML, FMM), World Federation of Societies of Anaesthesiologists (WFSA) Education Committee, https://wfsahq.org/about/people/committees/education-committee/ (DO), See attached list for the affiliations of the investigators of the Utstein Simulation Study Group (USSG)

## Abstract

**BACKGROUND:**

Despite its importance in education and patient safety, simulation-based education and training (SBET) is only partially or poorly implemented in many countries, including most European countries. The provision of a roadmap may contribute to the development of SBET for the training of anaesthesiologists.

**OBJECTIVE:**

To develop a global agenda for the integration of simulation into anaesthesiology specialist training; identify the learning domains and objectives that are best achieved through SBET; and to provide examples of simulation modalities and evaluation methods for these learning objectives.

**DESIGN:**

Utstein-style meeting where an expert consensus was reached after a series of short plenary presentations followed by small group workshops, underpinned by Kern's six-step theoretical approach to curriculum development.

**SETTING:**

Utstein-style collaborative meeting.

**PARTICIPANTS:**

Twenty-five participants from 22 countries, including 23 international experts in simulation and two anaesthesia trainees.

**RESULTS:**

We identified the following ten domains of expertise for which SBET should be used to achieve the desired training outcomes: boot camp/initial training, airway management, regional anaesthesia, point of care ultrasound, obstetrics anaesthesia, paediatric anaesthesia, trauma, intensive care, critical events in our specialty, and professionalism and difficult conversations. For each domain, we developed a course template that defines the learning objectives, instructional strategies (including simulation modalities and simulator types), and assessment methods. Aspects related to the practical implementation, barriers and facilitators of this program were also identified and discussed.

**CONCLUSIONS:**

We successfully developed a comprehensive agenda to facilitate the integration of SBET into anaesthesiology specialist training. The combination of the six-step approach with the Utstein-style process proved to be extremely valuable in supporting content validity and representativeness. These results may facilitate the implementation and use of SBET in several countries.

**TRIAL REGISTRATION:**

Not applicable.

**KEY POINTS**

- We used Kern's six-step approach and the Utstein-type process to develop a comprehensive program to facilitate the integration of simulation-based education into anaesthesiology specialist training.
- We identified ten specific domains for which simulation should be used: boot camp/initial training, airway management, regional anaesthesia, point of care ultrasound, obstetrics anaesthesia, paediatric anaesthesia, trauma, intensive care, critical events in our specialty, and professionalism and difficult conversations
- For each domain, we developed a course template that defines the learning objectives, the instructional strategies (including simulation modalities and simulator types), and assessment methods.
- To facilitate implementation and sustainability, local, national and global political and administrative leaders must be involved through ongoing and committed financial support.

## Background

Simulation based education and training (SBET) offers several advantages over traditional training methods. It provides unique opportunities to learn safely and ethically without risks and consequences for patients.^1^ From an educational perspective, learning is facilitated through authentic and contextualised experiences, feedback and reflection. It can be used effectively for individuals or teams to learn, practice, or assess technical and non-technical skills in a safe and controlled environment.^2–5^

There has been an impressive increase in the availability and diversity of simulated patients, manikins, part-task trainers, computer-based simulations, virtual reality, and augmented reality simulators. Today, these simulation modalities and simulator types can be used either alone or combined during hybrid simulation to target different learning objectives.^6^

Recently, the Lancet Commission recognised the wide application of information technology, including simulation, as one of the most transformative developments with the potential to have a lasting impact on health professional education, along with interprofessional and competency-based education.^7^ In 2020, the European Society of Anaesthesiology and European Section/Board of Anaesthesiology endorsed the principles of competency-based medical education and identified SBET as a key factor in its implementation.^8^ Anaesthesiologists were early adopters of modern simulation and have been leaders in developing simulation training for several decades.^9,10^ Nowadays regular simulation training is ranked among the top five anaesthesia-related safety practices in Europe.^11^

Nevertheless, despite its popularity and importance, not all anaesthesia trainees have access to SBET. A recent survey conducted among European national anaesthesiology societies found that only a few have formally integrated SBET into their residency training curricula.^12^ Survey respondents expressed a strong need for standards and recommendations, as well as assistance in developing a curriculum for high-fidelity simulation, areas in which international scientific societies have a clear role to play.^12,13^ As many countries have adopted the European Training Requirements in anaesthesiology,^14^ which share numerous similarities with North-American competency frameworks, international collaboration initiatives are clearly needed to facilitate the use of SBET.^15^ This could also help to improve the level of compliance with the training requirements, which remains heterogeneous across European countries.^16^

Developing a generic framework for a SBET programme could facilitate the implementation and use of simulation in anaesthesiology specialist training. This article reports on the consensus process (i.e., an Utstein-style meeting^17^), presents the results and discusses the findings of developing such a programme.

## Objectives

The overall goal was to develop a global agenda to integrate SBET into anaesthesiology specialist training. The specific objectives were to: identify the learning objectives that are best achieved through SBET, describe how to integrate SBET into anaesthesiology specialist training, provide examples of simulation modalities for specific learning objectives, and identify barriers and facilitators to implementation.

## Methods

### Ethics

Ethics review was not required since the study did not include patient or human data.

### Study design

The study design used a combination of a pre-meeting questionnaire and an Utstein-style meeting to develop consensus. Similar Utstein initiatives have been used to define a research agenda for SBET in healthcare and to identify topics that simulation can address to improve patient safety.^18,19^ Methods were consistent with the Utstein methodology used to develop agreement on a given topic.^17^

### Panel selection

The organising committee (GLS, CLB, ML, FMM, DØ) identified an international group of SBET experts involved in the training of residents at national levels, key members from the European Society of Anaesthesiology and Intensive Care (ESAIC), the Society for Simulation in Europe (SESAM) and the World Federation of Societies of Anaesthesiologists (WFSA). All participants had experience with the development of SBET and research. The list was established based on relevance of expertise and diversity in terms of countries.

A total of 25 participants were invited, two of whom were residents as representatives of the target audience. Most of the participants came from Europe, but there was geographical representation from North America, South America, and Africa. Several had experience with SBET outside their country. The names and countries of origin of the participants are available in Appendix 1 as supplemental digital content (SDC), and all are collaborators of this publication.

### Preparation of the Utstein meeting

Prior to the meeting, participants were informed of the objectives, process and expected outcomes, and were provided with relevant literature.^12,13,15,17^ They completed an 11-question survey on the use of SBET during residency training in their country (SDC, Appendix 2). Participants were asked to propose the top ten critical scenarios and top ten technical procedures for which all residents should be trained using SBET during their residency. This survey was a simplified and modified version of the recent European survey.^12^

### Format of the Utstein Meeting

The meeting took place in Copenhagen, Denmark, on 23–24 September 2022 (detailed programme available as SDC, Appendix 3). The meeting format consisted of a series of short plenary presentations followed by small group workshops in which participants were divided into three different groups. During these workshops, the groups were asked to refine and develop key concepts which were then presented in plenary to reach consensus. All plenum discussions were audio-recorded and a dedicated person took notes. Electronic or flip charts presentations were collected and copied. The meeting was followed by email correspondence among all the panel participants, and virtual meetings to resolve ongoing issues.

### Theoretical framework underpinning the meeting process

The programme outline and the progression of the meeting followed an adaptation of Kern's six-step approach to curriculum development.^20^

Step 1 – problem identification and general needs assessment: There is a need to provide standards, recommendations and assistance to develop and implement SBET during speciality training in anaesthesiology in Europe.^12^ This was briefly summarised during the inaugural presentations, which also included an outline of the Utstein consensus meeting and examples of outcomes of some previous meetings provided by Tore Laerdal, Executive Director of the Laerdal Foundation and Chairman of Laerdal Medical.

Step 2 – targeted needs assessment: The objectives of the participants’ survey were to understand each other's background and context and to gather expert opinions before selecting the priority educational areas that should be discussed at the meeting. A brief summary of the survey answers was presented, and based on the results, we decided to design a 30-day SBET curriculum distributed over a 5-year training period.

Step 3 – goals and objectives: The first working session prioritised areas of professional expertise for which SBET should be used to achieve training outcomes. The goal was to reach a consensus list on a limited number of ‘high-priority domains’. During the second breakout session, participants worked to define the learning objectives for each domain for which SBET should be used (i.e., knowledge application, practical/clinical skills, and specific attitudes). Within each domain, these objectives should focus on what is ‘difficult to learn and to assess’ and/or ‘what can harm the patient’.

Step 4 – educational strategies: Content and the educational methods were defined during the third working session. Group discussions were preceded by a brief presentation of the tools available for designing a comprehensive course on a given topic, as well as a generic course outline covering the ‘learning objectives’, ‘instructional strategies’, ‘equipment’, ‘formative assessment methods’ and ‘measurable learning outcomes’. The participants were asked to propose simulation modalities and simulators adapted to the objectives identified in Step 3 based on a list described elsewhere.^21^ In addition, they were asked to provide examples of how courses could be planned over the course of the 5-year programme and what other learning activities (refresher courses, blended learning, e-learning) could facilitate knowledge and skills transfer into the clinical setting.

Step 5 – implementation: This step involved identifying the facilitators of implementation and barriers to be addressed. Practical aspects such as the number of trainees and faculty, resources, finances, equipment, and political support were addressed using a SWOT analysis (Strengths, Weaknesses, Opportunities, Threats) during small group workshops.^22^

Step 6 – evaluation and feedback: A brief presentation covered the concepts of programme evaluation (Kirkpatrick) and learner evaluation (Miller's pyramid), as well as practical examples of how knowledge, skills, and attitudes can be assessed (formative or summative) using MCQs, e-learning, checklists, Objective Structured Clinical Examination (OSCE) and workplace-based assessments, whether at the end of a course or training. The group work consisted of proposing the integration of these elements into the courses developed on the first day.

After the meeting, the organising committee met on-site on the 28 November 2022 to analyse and edit all the materials that were produced in a structured and comprehensive way. During this meeting, the structure of the manuscript was defined, subsequent work was divided between the authors. On the 29 March 2023, a first version of the article was sent to all participants for critical review. Further changes were made, and the final version of the manuscript was submitted on May 25th.

## Results

### Targeted needs assessment – survey results

All participants answered the survey, of the 22 countries represented ‘anaesthesia’ and ‘critical care’ were combined into one medical specialty in 14 countries, including in 13 European countries out of 16. SBET was well implemented in 11 countries and to some extent in 11 countries. A wide variety was observed in the total number of high-fidelity simulation training days offered to trainees: ranging from <10 days in 43% of countries to between 10 and 50 days in 57%. Twelve countries used SBET for formative assessment and six for summative assessments.

Participants’ suggestions for the top ten critical scenarios and the top ten technical procedures for which all residents should be trained using SBET were grouped into themes/areas (Table 1). The scenarios were related to crisis situations and covered both medical expertise and non-technical skills defined as social and cognitive skills such as communication, collaboration, leadership/followership, situation awareness and clinical decision-making. Specific situations, such as anaphylactic shock, malignant hyperthermia, obstetric and paediatric crises, were mentioned. These examples illustrate situations in which we cannot prepare trainees in the clinical setting because they are opportunistic and cannot be planned for. The procedures were within the categories: airway, vascular access, and regional block. Ultrasound-guided procedures and point-of-care ultrasound (POCUS) were also mentioned.

**Table 1:** Top 10 scenarios and procedures for which all trainees should be trained. Numbers and percentages indicate the number of times and proportions participants cited a given scenario or procedure

Critical clinical scenarios		Number	%
Crisis situations	Handling the difficult airway	23/23	100%
	Anaphylaxis	21/23	91%
	Malignant hyperthermia	15/23	65%
	Cardiac arrest	15/23	65%
	Local anaesthesia toxicity, high block	6/23	35%
	Cardiogenic Shock, Embolus	5/23	22%
Specific patient groups	Obstetrical crisis situations	14/23	61%
	Trauma and massive bleeding	10/23	43%
	Crisis situations in paediatrics	9/23	39%
Adverse events	Disclosure and debriefing of team	6/23	26%

Procedures		Number	%
Airway	Basic	19/23	83%
	Advanced	19/23	83%
	Cricothyroidotomy	15/23	65%
Vascular access	Central line	23/23	100%
	Arterial line	9/23	39%
	Peripheral vein	7/23	30%
	Intraosseous	5/23	22%
Nerve blocks	Central nerve blocks	22/23	96%
	Peripheral nerve blocks	14/23	61%
Point of care ultrasound	Heart, lung, abdomen, etc.	15/23	65%

### Goals – identifying high-priority domains

After the first morning sessions, consensus was reached on a curriculum covering the following ten high-priority domains: Boot camp/initial training, Airway management, Regional anaesthesia, Point of care ultrasound, Obstetric anaesthesia, Paediatric anaesthesia, Trauma, Intensive care, ‘Critical events in our specialty’, and ‘Professionalism and difficult conversations’. We agreed to include the first four domains, but decided not to cover them in detail during the meeting because many courses already exist on these topics. In order to save time this was done after the meeting.

### Learning objectives, educational strategies, assessment and evaluation

The following sections summarise the consensus process. Each section highlights what is specific to each of the ten high-priority domains. Learning objectives, course length, instructional strategies and assessment methods are summarised in a SBET course template, which is provided in a table or appendix (available as SDC). For each course the focus was on learning and the experts stressed the importance of respecting the basic principles of SBET to ensure learners’ psychological safety.^23–25^ Confidentiality, getting trainees to know each other if they come from different departments, focusing on improvement, and building down from a course using learning plans and work-based assessments all contribute to these goals.

#### Bootcamp/initial training

A novice anaesthesiology trainee can benefit from participation in a 3- to 5-day bootcamp at the start of training.^26^ Residents are introduced to equipment, medications as well as the most common procedures and the necessary social and cognitive skills. A combination of interactive lectures, hands-on skills training and simulation scenarios followed by debriefing can provide them with the knowledge and skills needed to provide safe patient care (see SDC, Appendix 4). This can be done in a safe environment where skills can be trained until the technique is mastered.^27,28^ In some countries, all trainees start once or twice a year, making it easier to plan the training, while in other countries, trainees may start monthly. In this case, collaboration between several departments/hospitals can be helpful.

#### Airway management

An airway management course should consist of blended content and encompass a variety of educational strategies, methods, and tools. It was emphasised that acquiring and maintaining airway management skills is critical and that airway training should begin early during the bootcamp and extend over several days, possibly followed by annual refresher courses. Table 2 presents a template for an airway course that integrates SBET. The user may select the learning objectives relevant to a particular training stage, that is, starting with basic airway training for beginners followed by advanced training in airway management for more senior trainees. Table 2 also includes a selection of useful references, generic to SBET and applicable to other domains,^23–25,29,30^ and others specific to airway management.^31–37^

**Table 2:** SBET airway management course

Overall learning goals
Provide all anaesthesiology residents with structured training in the equipment and techniques most frequently used during routine clinical practice and advanced AWM
Develop and practice the social and cognitive skills relevant to AWM

Learning objectives	Examples of educational strategies and tools	Methods of formative evaluation and measurable learning outcomes
Knowledge		
Normal airway anatomy and raise awareness of difficult airway anatomy.	Interactive lectures	Pre- and post-MCQs
Oxygenation and ventilation physyiology; methods for optimising oxygenation, improve ventilation and extending apnoeic time	E-learning	Formative assessment
General and specific pharmacology relevant to AWM, e.g. NMBA, reversal agents in anaesthesia	Guidelines/selected publications^23–25,30^	
Monitoring techniques, e.g. pulse oximetry, capnography	Website of difficult airway societies	
Airway assessment	Selected social media resources	
Airway rescue techniques, how to anticipate and plan for and manage a difficult airway		
Cognitive aids (e.g. AWM guidelines)		
Poor AWM outcomes and deficiencies related to judgement, communication, planning, equipment and training		

Clinical skills	Examples of hands-on clinical skills stations using part-task trainers	
To develop basic and advanced airway skills (following a longitudinal competence-based training curriculum)	Face mask ventilation techniques; direct and videolaryngoscopy; SAD insertion and intubation via SAD; Flexible Bronchoscopy; HFNO; e-FONA techniques; Lung isolation techniques	OSCE (checklist, global rating scale) video assessment (self, peers, faculty)
To familiarise with and practice using equipment and techniques commonly used in clinical practice	Video-assisted demo of rescue techniques	Work-based assessment during routine cases (DOPS)
To familiarise with and practice guidelines relevant to each institution/region/country	Expert live demonstration	Peer and expert feedback
To familiarise with and practice rescue airway management protocols and guidelines^31–37^		Portfolios

Simulation for applying knowledge, clinical skills and social and cognitive skills (non-technical)	Examples of HF simulated airway scenarios followed by debriefing	
To integrate knowledge, airway skills, and social/cognitive skills in the management of simulated airway crisis	Unanticipated difficulties in routine AWM	Structured reflective debrief by trained faculty^23–25,30^
To develop social and cognitive skills, e.g., anticipation and planning, task management, communication and team working, decision-making and situation awareness	Unanticipated difficulties in rapid sequence induction	ANTS taxonomy^29^
To understand the role of human factors and ergonomics in airway crisis^37^	Intraoperative hypoxia secondary to airway devices displacement/blockage	Learning plans
To train together with other specialities (e.g. general surgery, emergency medicine, otorhinolaryngology residents) and understand their roles	Displaced tracheostomy	Work-based assessment
To encourage reflection on one's own and peers’ performance in a safe simulation environment	Unrecognised oesophageal intubation	
To learn how to receive and give constructive feedback aimed at reflection and learning	Cannot intubate cannot oxygenate & eFONA	
To devise learning plans to bring home to the training supervisor	Difficult/failed extubation	

ANTS, anaesthetists’ non-technical skills; AWM, airway management; DOPS, direct observation of procedural skills; e-FONA, emergency front of neck access; HF, high-fidelity; HFNO, high-flow nasal oxygen; MCQ, multiple choice questions; NMBA, neuromuscular blocking agents; OSCE, objective structured clinical examination; SAD, supraglottic airway devices.

#### Regional anaesthesia

Acquiring skills in regional anaesthesia including ultrasonography has become a mandatory component of modern training in anaesthesiology. Foundation training should aim at learning and deliberate practice of a small number of versatile techniques that cover the vast majority of surgical procedures and therefore provide patient access to reliable and safe regional anaesthesia.^38,39^ Competence in the more advanced blocks should be acquired during advanced fellowship in regional anaesthesia. Appendix 4 (SDC) provides a template for a regional anaesthesia SBET course that incorporates these principles. Of note, the specific number of repetitions to achieve proficiency in a certain type of nerve block continues to be the subject of much debate and varies from one national training programme to another.^39^

#### Point of care ultrasound

The importance of Point of care Ultrasonography (POCUS) is increasingly recognised in our speciality and is part of the modern curriculum.^14^ Using bedside ultrasonography for diagnostic and therapeutic purposes helps improve perioperative care. SBET can significantly enhance the knowledge and practical skills in POCUS testing.^40^ Appendix 4 (SDC) provides a template for such a course.

#### Obstetrics anaesthesia

The panel emphasised the importance of using SBET to allow anaesthesia trainees the opportunity to develop technical and social-cognitive skills in managing simulated obstetric crises. It is fundamental that part of this training be conducted during interprofessional simulations with midwives, nurses, obstetricians, and paediatricians. This can be achieved through in situ simulated exercises or in a simulation centre. Several studies have shown that this approach has a positive impact on patients’ management.^41,42^Table 3 details the template of a three-day course that can be distributed according to training level.

**Table 3:** SBET obstetric anaesthesia course

Overall learning goals
Provide all anaesthesiology residents with structured training in routine obstetric anaesthesia and in critical situations
Develop and practice social and cognitive skills relevant to interprofessional collaboration and multi-disciplinary teamwork in the labour and delivery room

Learning objectives	Examples of educational strategies and tools	Methods of formative evaluation and measurable learning outcomes
Knowledge		
Management of parturient during normal labour	Pre-course (half-day preparation):	Pre- and post-MCQs
Management of parturient during caesarean section	Book reading	Serious games online
Management of parturient during obstetric emergencies	E-learning (e.g. ESAIC Academy)	Feedback from scores
Surgery in pregnant women for non-obstetric causes	Interactive lectures/case-based discussion	

Clinical skills	Examples of hands-on clinical skills stations using part-task trainers	
Loco-regional anaesthesia skills: spinal/epidural/combined?, TAP block, ultrasound techniques	Part-task trainer, ultrasound live models, VR	Checklist, global rating scale
Management of accidental dural puncture	Part-task trainer, Screen-based simulation, VR	Peer and expert feedback
Specificities of airway management skills in obstetric patients	Part-task trainer	Work-based assessment during routine cases (DOPS)
Maternal cardiopulmonary resuscitation	Low or high fidelity full scale manikin	Portfolio
Breaking bad news	Role play	

Simulation for applying knowledge, clinical skills and social and cognitive skills (non-technical)	Examples of HF simulated obstetrical scenarios followed by debriefing	
To integrate knowledge, technical skills, and social/cognitive skills in the management of simulated OB crisis	Conversion of Spinal to GA	Structured reflective debrief by trained faculty^23–25,30^
To train together with obstetricians and midwife and understand their roles and responsibilities	Emergency caesarean section under GA	Observation of teamwork skills
To develop and practice interprofessional collaboration and multi-disciplinary teamwork	Difficult airway during caesarean section	Medical/crisis checklists
To improve team communication, shared situational awareness and decision making in critical OB situations	Pre-eclampsia/eclampsia/HELLP	
	PPH and massive transfusion protocol	
	Amniotic fluid embolism including maternal cardiac arrest and perimortem caesarean section	
	Total spinal	
	Maternal sepsis	

DOPS, direct observation of procedural skills; ESAIC, European Society of Anaesthesiology and Intensive Care; GA, general anaesthesia; HELLP, haemolysis, elevated liver enzymes, low platelets; OB, obstetrics; PPH, postpartum haemorrhage; TAP, transverse abdominis plane; VR, virtual reality.

#### Paediatric anaesthesia

A 2- to 4-day course of paediatric anaesthesia was described. It can be conducted in one course or divided in a basic and a more advanced course. Appendix 4 (SDC) gives examples of the learning objectives and methods used for learning and assessment. Overall, basic skills are related to a child with a normal anatomy/physiology and more advanced skills relate to more difficult cases/situations/complications. Educational strategies include pre- and in-course tools, as well as suggested formative assessment tools for use immediately after simulations or in the clinical setting (work-based assessment).

#### Trauma

Appendix 4 presents a template for the application of SBET in a trauma course. The management of trauma patients requires distinct skills, which are performed using a coordinated multi-disciplinary approach. This course aims to train the assessment, resuscitation, and perioperative care of patient with critical trauma. In addition, scenarios should highlight time pressure in a highly dynamic context and offer opportunities to train social and cognitive skills.

#### Intensive care

Although critical care medicine requires separate certification in some countries, it remains a core competence of anaesthesiologists in all European countries.^14,43^ Appendix 4 (SDC) provides a template for a 3- to 4-day SBET course highlighting key technical procedures, critical clinical scenarios, multi-disciplinary teamwork, interprofessional collaboration and interaction with patients’ relatives.

#### Critical events in our specialty

Critical events in anaesthesiology are uncommon but have great potential for harm. SBET is widely used to improve the knowledge, medical expertise, and social and cognitive skills required to manage critical situations.^2,3,12^. Training should focus on early recognition and treatment of specific events to improve safety in the operating room. The template of this course is presented in Table 4.

**Table 4:** SBET critical events course

Overall learning goals
Provide all anaesthesia residents with formal training in the management of critical events in the perioperative setting.
Develop and practice social and cognitive skills in the context of critical anaesthesia events

Learning objectives	Examples of educational strategies and tools	Methods of formative evaluation and measurable learning outcomes
Knowledge		
Airway and respiratory emergencies management	Pre-course (one-day preparation):	Pre- and post-MCQs
Diagnosis and treatment of cardiac events	Anaesthesia crisis manual/textbook	Case-based discussion
Identification of adverse events related to regional anaesthesia	Institutional cognitive aids/checklists/protocols	
Comprehend the aetiology and approach of drug related adverse events	E-learning (e.g. ESAIC Academy)	
Procedures in case of fire and equipment failure	Real institutional critical incidents	

Clinical skills		
Variable depending on the critical event that will be trained	Screen-based simulation	Performance metrics during screen-based simulated crisis management
Ensure that the learner's knowledge and clinical skills are appropriate for the scenario	Part-task trainer	Work-based assessment during routine cases (DOPS)
	Case-based discussion for knowledge application	Portfolio

Simulation for applying knowledge, clinical skills and social and cognitive skills (non-technical)	Examples of HF critical simulated scenarios followed by debriefing	
To rapidly recognise and manage a critical incident using appropriate cognitive aids	Anaphylaxis	Structured reflective debrief by trained faculty^23–25,30^
To utilise adequate resources and equipment	Malignant hyperthermia during laparoscopy	Observation using medical/crisis checklists
To perform a differential diagnosis in unexpected and dynamic situations	Cardiac arrest after induction	Behavioural global rating scale, e.g., ANTS^29^
To practice dynamic situation awareness and continuous reassessment	Elevated airway pressure	
To practice decision making, task management, leadership and effective communication	Can’t ventilate, can’t oxygenate	
	Local anaesthetic systemic toxicity	
	Cardiogenic shock	
	Pulmonary embolus	
	Fire in the operating room	

ANTS, anaesthetists’ non-technical skills; DOPS, direct observation of procedural skills; ESAIC, European Society of Anaesthesiology and Intensive Care; MCQ, multiple choice questions.

#### Professionalism and difficult conversations

This section covers a wide variety of challenging situations that reflect both the professional and communicator roles. These situations can be trained using standardised patients/actors in role-plays or simulations, followed by debriefing and reflection. A two- to three-day course focusing on specific situations would be beneficial (SCD, Appendix 4), whereas some could be trained in relation to other courses, such as paediatric, airway or critical events courses.

### Implementation of simulation-based education and training

The SWOT analysis of the practical implementation and the blockers and facilitators of such programmes are presented in Tables 5 and 6, respectively. We realised that many strengths can also be weaknesses and vice versa. For example, support from international societies (e.g., ESAIC, SESAM) can be a valuable asset, but it can also be perceived as externally imposed changes. Similarly, aspects of resources (space, personnel, equipment, simulators) can be significant challenges or become assets (pre-existing space, increased professional recognition, collaborations with industry).

**Table 5:** SWOT analysis regarding SBET implementation

Strengths	Weaknesses
• Options of partial self-sustainability through external courses	• Shared spaces
• Being/building up a centre of excellence	• Human resources
• Existing human resources	• Training of instructors, quality assurance
	• Necessity of approaching higher political and administrative bodies
	
Opportunities	Threats
• New fields of work	• Shortage of personnel, overwhelming by other duties
• Research	• Financial difficulties
• Administrative support	• Active opposition by societies/politics/administration
• Upcoming new, maybe AI, tools	

**Table 6:** Summary of barriers/blockers and supporters/facilitators to SBET implementation

	Barriers/blockers	Supporters/facilitators
Local	• Resistance to change	• Interest in training, becoming part of the team
	• Faculty time	• Buy-in of local leadership
	• Need for space	• Foundations
	• Need for funding	
National	• Not recognising need for change	• Accreditation standards
	• Lack of outcome measures	• Patient organisations
	• Need for funding	• Embedded outcome measures
		• National grants
Global	• Perception of external initiative/influence	• Global support by supranational organisations (ESAIC, SESAM)
	• (Wrong) perception of safety level	• Technological advances
		• Research evidence

The need for continued and committed financial support was identified as a major prerequisite for sustainability. There was a broad consensus among experts that a number of key factors need to be taken into account in order to succeed. First, local, national, and global political and administrative leaders must be involved quickly. To gain support, it may be useful to focus on patient safety as advocated or mandated by local or broader regulations and to broaden the focus to accreditation and certification bodies. Ideally, appropriate outcome measures should be planned and incorporated into the program structure. Second, grants can help get the program off the ground; however, sustainability must be planned from the start. Third, this also applies to staffing and careful planning for teacher training, identification of future instructors and strong internal processes (e.g., case library, rotation of materials, recognition of time use), which are necessary to ensure durability. Finally, depending on local circumstances, different approaches are required for curriculum planning.

## Discussion

This Utstein consensus process has defined a SBET agenda for anaesthesiology, focusing on areas of our specialty where simulation can make a difference and significantly support learning. The experts identified ten priority domains for which simulation is particularly relevant. These domains can be considered as the *‘simulation zone*’ for anaesthesiology, a concept described earlier that refers to tasks that are either difficult to learn (due to their low frequency or particular difficulty) and/or due to their high stakes (i.e., their potential to expose patients to serious risks).^44^ For each domain, we developed a SBET course template that include learning objectives, simulation modalities and types of simulators, and suggested assessment methods. This represents the first generic roadmap for implementing SBET in anaesthesiology specialist training. This educational agenda could help fill the gaps identified in the recent European survey, which found that 43% of countries did not have the resources to develop a curriculum and 60% wanted access to a detailed curriculum for high-fidelity simulation.^12^

European and North American, postgraduate anaesthesiology competency frameworks share many similarities.^15^ This supports the generalisation of our results. However, in Europe, although many countries have adopted the ETR in anaesthesiology,^14^ there are still differences in the duration of postgraduate training, assessment methods and certification requirements.^16,45^ Furthermore, ‘anaesthesia’ and ‘intensive care’ can be combined into one medical specialty or certified separately depending on the country. The proposed educational agenda should be adapted and tailored to national and local contexts. When implementing a new national SBET curriculum, the priority should be given to content that is of utmost importance to the needs and practices of the specialty in this country.

Simulation offers unique opportunities to help learners develop their skills and assess their progress. It is well established that, aligning the learning objectives, the teaching strategies and the assessment formats is a powerful driver for learning.^46^ To promote implementation, SBET programmes must be aligned with the residency curriculum and the final examinations. We have paid particular attention to the coherence between the proposed programme and the training requirements, however the question of alignment with the certification exam remains open.

The number of training days allocated to each domain as well as the focus on certain learning objectives should be guided by the current safety issues in perioperative medicine. To be successful, strategic implementation and careful curriculum planning should consider several local variables and specificities, some of which are highlighted in our results. For example, if all new trainees start on the same date there will be a spike in activity and demand for space, but planning will be easier. In contrast, if new trainees arrive at any time, more careful planning is required but space and staffing requirements for training are easier to meet. Further guidance can be found in the Irish ‘National Simulation Strategic Guide for the Implementation of Simulation on Clinical Sites’.^47^

Overlaps between the domains in terms of medical content and learning objectives are inevitable. For example, a difficult airway scenario naturally belongs in the *“airway course”* but it could also be contextualised in *“obstetrics”*, *“paediatrics”* or in the “*critical events in our specialty”* courses. Similarly, generic social and cognitive skills such as communication, collaboration, leadership, situational awareness, and clinical decision-making should be included in all domains. Some repetition is not a problem in itself, on the contrary, when properly planned in a spiral curriculum, iterative revisions of important concepts contribute to competence development.^48^

The content validity and representativeness of our results were strengthened by the methods used. First, we developed this agenda according to a well accepted theoretical framework in medical and anaesthesiology education, namely Kern's six-step approach,^20,49–51^ which allowed us to address all aspects of the curriculum systematically. Second, we invited 25 participants, a number in accordance with previous Utstein meetings to offer a reasonable balance between efficiency and inclusiveness.^17^ Most participants came from the same geographical region (Europe), and while many had previous experience of SBET activities in countries other than their own, this may have influenced the results and overall generalisation. Another possible limitation is that two-thirds of these European countries were in Western, Central or Northern Europe. Nonetheless, different continents and countries were represented, including representatives of the education committee of the WFSA. Although the results represent a consensus more readily applicable to high-income countries, they can also be adapted to low- and middle-income countries, as demonstrated by the publication of successful targeted SBET programmes.^51–55^

As the main objective was to provide a generic framework to facilitate the implementation of an SBET programme, the detail of the information remains limited in terms of timetable and teaching sequence of the various domains. Similarly, the description of the important ‘high-fidelity scenarios’ was limited to their title and we did not provide a scenario template or develop examples of high-fidelity simulation scenarios. However, examples of templates^47,56^ and detailed critical anaesthesia scenarios are readily available (https://www.fhft.nhs.uk/careers/clinical-education/quest-home/simulation-scenarios/). Finally, participants repeatedly stressed the importance of ensuring faculty development in simulation. We could not elaborate on this, but there are useful resources on the subject, such as the Irish Guide mentioned above.^47^

## Conclusion

We successfully developed a comprehensive agenda to facilitate the integration of SBET into anaesthesiology specialist training. The combination of the six-step approach with the Utstein-style process proved to be extremely valuable in supporting content validity and representativeness. These results may facilitate the implementation and use of simulation in several countries.

## Supplementary Material

Supplemental Digital Content

## Supplementary Material

Supplemental Digital Content

## Supplementary Material

Supplemental Digital Content

## Supplementary Material

Supplemental Digital Content
