# Supplementary material for: Integration of simulation-based education in anaesthesiology specialist training: Synthesis of results from an Utstein Meeting
Source: Eur J Anaesthesiol. 2023 Oct 19;41(1):43–54. doi: 10.1097/EJA.0000000000001913 (PMC10720798; doi:10.1097/EJA.0000000000001913)
Supplement: Supplemental Digital Content [file ejanet-41-43-s001.docx]

**Utstein Simulation Study Group – Authors’ Names and affiliations** (alphabetical order; *Organizing committee members)

| **Last name** | **First name** | **Country** | **ORCID** | **Affiliation** | **Email address** |
| --- | --- | --- | --- | --- | --- |
| Borre Jacobsen | Rikke Vita | Denmark | N/A | Department of Clinical Medicine, Anaesthesiology, University of Copenhagen, Copenhagen, Denmark | [Rikke.Vita.Borre.Jacobsen@regionh.dk](mailto:Rikke.Vita.Borre.Jacobsen@regionh.dk) |
| Brzezinski Sinai | Yitzhak | Israel | 0000-0001-6312-4113 | Division of Anaesthesia, Intensive Care and Pain Medicine, Tel Aviv Medical Centre, Tel-Aviv, Israel | [isaac.sinai@gmail.com](mailto:isaac.sinai@gmail.com) |
| Buleon | Clément | France | 0000-0003-4550-3827 | Anaesthesiology, Perioperative and Pain Medicine, Polyclinique du Parc, Caen, France  University Hospital Liège, Belgium  University Hospital Caen-Normandy, France  Center for Medical Simulation, Boston, MA-USA | [clement.buleon@wanadoo.fr](mailto:clement.buleon@wanadoo.fr) |
| Burlacu * | Crina L. | Ireland | 0000-0001-9309-5544 | Department of Anaesthesia, Intensive Care and Pain Medicine, St. Vincent's University Hospital, Dublin, Ireland | [crina.burlacu@ucd.ie](mailto:crina.burlacu@ucd.ie) |
| Chiu | Michelle | Canada | 0000-0002-0661-1326 | Department of Anesthesiology and Pain Medicine, The Ottawa Hospital and University of Ottawa, Ottawa, Canada | [mchiu@toh.ca](mailto:mchiu@toh.ca) |
| Corvetto | Marcia | Chile | N/A | Department of Anesthesiology, Escuela de Medicina, Pontificia Universidad Católica de Chile, Santiago, Chile; Experimental Surgery and Simulation Center, Department of Digestive Surgery, Escuela de Medicina, Pontificia Universidad Católica de Chile, Santiago, Chile | [marciacorvetto@gmail.com](mailto:marciacorvetto@gmail.com) |
| Creutzfeldt | Johan | Sweden | 0000-0001-9406-8484 | Center for Advanced Medical Simulation and Training and Department for Clinical Sciences, Intervention and technology, Karolinska University Hospital and Karolinska Institutet, Stockholm, Sweden | [Johan.Creutzfeldt@ki.se](mailto:Johan.Creutzfeldt@ki.se) |
| Karmelić | Dora | Croatia | 0000-0003-2328-7764 | Clinic of Anesthesiology, Reanimatology, Intensive Medicine and Pain therapy, University Hospital Centre Zagreb, Zagreb, Croatia | [dora.karmelic@gmail.com](mailto:dora.karmelic@gmail.com) |
| Khalid | Karima | Tanzania | 0000-0001-9917-9853 | Department of anaesthesia, Muhimbili University of Health and Allied Sciences. Department of anaesthesia, Muhimbili Orthopaedic Institute. Dar es Salaam, Tanzania | [karimakhalid@yahoo.com](mailto:karimakhalid@yahoo.com) |
| Krage | Ralf | UAE | 0000-0002-7024-2633 | Khalaf Ahmad Al Habtoor Medical Simulation Center. Mohammed Bin Rashid University of Medicine and Health Sciences. Dubaï, UAE. | [krageralf@gmail.com](mailto:krageralf@gmail.com) |
| Lazarovici * | Marc | Germany | 0000-0003-2694-810X | Institute for Emergency Medicine and Management in Medicine, Ludwig Maximilians University Hospital, Munich, Germany | [marc.lazarovici@med.uni-muenchen.de](mailto:marc.lazarovici@med.uni-muenchen.de) |
| Lilaonitkul | Maytinee | USA | 0000-0001-8420-9589 | Department of Anesthesia and Perioperative care, University of California San Francisco, CA-USA | [m.lilaonitkul@gmail.com](mailto:m.lilaonitkul@gmail.com) |
| Lysgaard Poulsen | Jannie | Denmark | N/A | Copenhagen Academy for Medical Education and Simulation, Capital Region of Denmark and Department of Clinical Medicine, University of Copenhagen, Copenhagen, Denmark | [jannie.lysgaard.poulsen@regionh.dk](mailto:jannie.lysgaard.poulsen@regionh.dk) |
| Maio Matos * | Francisco | Portugal | 0000-0001-8968-3124 | Anaesthesiology Department, Centro Hospitalar e Universitário de Coimbra, CHUC, Coimbra, Portugal and Faculty of Medicine, University of Coimbra, FMUC, Coimbra, Portugal | [franciscomaiomatos@gmail.com](mailto:franciscomaiomatos@gmail.com) |
| Morten Mellemstrand-Paulsen | Pål | Norway | N/A | Department of Anaesthesia, Stavanger University Hospital, Stavanger, Norway | [pal.morten.mellemstrand-paulsen@sus.no](mailto:pal.morten.mellemstrand-paulsen@sus.no) |
| Novak-Jankovic | Vesna | Slovenia | 0000-0002-5786-1499 | Medical Simulation Centre  University Medical Centre  Ljubljana, Slovenia | [vnovakjankovic@gmail.com](mailto:vnovakjankovic@gmail.com) |
| Østergaard * | Doris | Denmark | 0000-0001-8542-6999 | Copenhagen Academy for Medical Education and Simulation, Capital Region of Denmark and Faculty of Medicine, University of Copenhagen, Copenhagen, Denmark | [doris.oestergaard@regionh.dk](mailto:doris.oestergaard@regionh.dk) |
| Petrisor | Cristina | Romania | 0000-0002-9140-9674 | “Iuliu Hațieganu” University of Medicine and Pharmacy, Cluj-Napoca, Romania | [indreicristinalaura@yahoo.com](mailto:indreicristinalaura@yahoo.com) |
| Savoldelli * | Georges L. | Switzerland | 0000-0002-8968-6920 | Division of Anaesthesia, Department of Anaesthesiology, Clinical Pharmacology, Intensive Care and Emergency Medicine. Geneva University Hospitals and Faculty of Medicine University of Geneva, Geneva, Switzerland | [georges.savoldelli@hcuge.ch](mailto:georges.savoldelli@hcuge.ch) |
| Sciberras | Stephen | Malta | 0000-0001-8852-4084 | Simulation Centre, Department of Anaesthesia, ITU & Pain Management, Mater Dei Hospital, Msida, Malta | [stephen.sciberras@gov.mt](mailto:stephen.sciberras@gov.mt) |
| Stourac | Petr | Czech Republic | 0000-0003-1944-5926 | Department of Simulation Medicine, Medical Faculty of Masaryk University and Department of Paediatric Anaesthesiology and Intensive Care Medicine, University Hospital Brno and Medical Faculty of Masaryk University, Brno, Czech Republic | [petr.stourac@gmail.com](mailto:petr.stourac@gmail.com) |
| [Tommila](mailto:Miretta.tommila@tyks.fi) | Miretta | Finland | 0000-0002-0798-4546 | Department of Perioperative Services, Intensive Care Medicine and Pain Management, Turku University Hospital and University of Turku, Turku, Finland | [miorli@utu.fi](mailto:miorli@utu.fi) |
| van Haaperen | Maartje | Netherlands | 0000-0002-0724-330X | Anaesthesiology Department, Amsterdam University Medical Centre and Faculty of Medicine, University of Amsterdam, UvA, Amsterdam, The Netherlands | [m.vanhaperen@amsterdamumc.nl](mailto:m.vanhaperen@amsterdamumc.nl) |
| Vlassakova | Bistra | USA | 000-003-3868-4535 | Department of Anaesthesiology, Critical care and Pain Medicine Boston Children’s Hospital/Harvard Medical School  Boston, MA-USA | [bistra.vlassakova@childrens.harvard.edu](mailto:bistra.vlassakova@childrens.harvard.edu) |
| Yazbeck Karam Abi Raad | Vanda | Lebanon | 0000-0001-8709-6778 | Anesthesiology Department, Lebanese American University- Medical Center-Rizk Hospital,  Beirut, Lebanon. | [vanda.abiraad@laumcrh.com](mailto:vanda.abiraad@laumcrh.com) |
