# Supplementary material for: Integration of simulation-based education in anaesthesiology specialist training: Synthesis of results from an Utstein Meeting
Source: Eur J Anaesthesiol. 2023 Oct 19;41(1):43–54. doi: 10.1097/EJA.0000000000001913 (PMC10720798; doi:10.1097/EJA.0000000000001913)
Supplement: Supplemental Digital Content [file ejanet-41-43-s002.docx]

# Appendix 2: Pre-Utstein meeting survey to participants

## General information and contact information of the responder:

First Name:

Last Name:

Country:

Role and responsibility in your National Anesthesia society:

## Preliminary notes:

- Please answer the survey questions based on your national context, curriculum and final board certification that is obtained at end of residency training (Anesthesiology or Anesthesiology and Intensive Care)
- Residency training = Postgraduate training = The training period after medical school that allows the Board speciality to be granted
- Resident - a physician, who is registered in a specialist training program in order to obtain the Board speciality
- Simulation-based education and training - the application of a simulation method or a simulator to enhance and facilitate teaching/training and learning.
- High Fidelity simulation refers to simulation experiences that are extremely realistic and provide a high level of interactivity and realism for the learner; Can apply to any mode or method of simulation; for example: human, manikin, task trainer, or virtual reality. (Ref: Sim dictionary)
- High Fidelity Simulators - a term used to refer to the broad range of full-body manikins that have the ability to mimic, at a very high level, human body functions. (Ref: Sim dictionary)
- Procedural simulation - the use of a simulation modality (for example, task trainer, manikin, computer) to assist in the process of learning to complete a technical skill(s), or a procedure, which is a series of steps taken to accomplish an end.
- Non-technical Skills encompass in the healthcare field, the skills of communication, (patient-provider, team) leadership, teamwork, situational awareness, decision-making, resource management, safe practice, adverse event minimization/mitigation, and professionalism; also known as behavioral skills or teamwork skills (ASSH). (Ref: Sim dictionary)
- Crisis resource management skills are in this survey considered as the non-technical skills, that are trained in the context of a simulated critical scenario
- Procedural / Technical skill is a skill that is required for the accomplishment of a specific task or procedure. In healthcare, the knowledge, skill and ability to accomplish a specific medical task or procedure; for example, inserting a chest tube or performing a physical examination.

*Definitions have been adopted or adapted from the following reference:* Healthcare Simulation Dictionary <https://www.ssih.org/Dictionary>

## Survey questions:

1. **In your country, specialty certification in anaesthesia and intensive care are (2 possible options to choose from):**

- Two separate medical specialist certifications
- Combined in one medical specialist certification

1. **Which situation would best describe the current state of simulation-based education during residency training in your country?**
2. It is well implemented at a National level and all Residents have access to it during their training
3. It is offered in large training centres and the majority of the residents have access to it during their training
4. It is only offered in some training centres and many residents do not have access to it during their training
5. It is currently marginal and access to it is very limited for Residents
6. It is not available
7. **Over the entire residency training (4–5-year program), what is the total number of training days during which High Fidelity simulations are used as learning methods (provide an estimate)?**
8. **Do you have any important information to provide us regarding access to simulation-based training during postgraduate training in your country?**
9. **For which learning objectives are High Fidelity simulations used during postgraduate training? (Thick every appropriate answer)**
   - To improve Residents’ non-technical/crisis resource management skills
   - To improve medical management of rare critical events (examples: anaphylaxis, difficulty airway, malignant hyperthermia, cardiac arrest in the operating room, ...)
   - For interprofessional/team training where anaesthesia residents learn together with participants from other professions/specialty (example: anaesthesia or operating room nurses, surgeons, obstetricians, ...)
   - To improve residents’ technical skills
   - Advanced life support skills development
   - Research
   - Provide any useful information regarding the coordination/organisation of these courses at the national level:
   - Other (please specify):
10. **List the top ten critical scenarios for which all residents should be trained using high-fidelity simulation during their postgraduate training** (ten answers expected)
11. **List the top ten technical skills/procedures for which all residents should be trained using procedural simulation during their postgraduate training** (ten answers expected)
12. **Do you have any important information to provide us regarding the use of High-Fidelity simulation-based training during postgraduate training in your country?**
13. **Are there activities planned before and/or after simulation-based educational activities/courses?**
14. **In your country, is simulation used as a formative assessment method during residency training? YES (please provide some information on how it is used) / No**
15. **In your country, is simulation used as a summative assessment method during residency and/or for the final national board examination? YES (please provide some information on how it is used) / No**
