# Supplementary material for: Integration of simulation-based education in anaesthesiology specialist training: Synthesis of results from an Utstein Meeting
Source: Eur J Anaesthesiol. 2023 Oct 19;41(1):43–54. doi: 10.1097/EJA.0000000000001913 (PMC10720798; doi:10.1097/EJA.0000000000001913)
Supplement: Supplemental Digital Content [file ejanet-41-43-s003.docx]

# Appendix 3. The program for the 2-days meeting

## Day 1 (2022.09.23):

## Day 2 (2022.09.24):
